# An Integrative Approach for the Characterization of Plant-Pathogenic *Streptomyces* spp. Strains Based on Metabolomic, Bioactivity, and Phylogenetic Analysis

**DOI:** 10.3389/fmicb.2021.643792

**Published:** 2021-03-22

**Authors:** Valentina Croce, Andrés López-Radcenco, María Inés Lapaz, María Julia Pianzzola, Guillermo Moyna, María Inés Siri

**Affiliations:** ^1^Laboratorio de Microbiología Molecular, Departamento de Biociencias, Facultad de Química, Universidad de la República, Montevideo, Uruguay; ^2^Programa de Posgrado de la Facultad de Química, Facultad de Química, Universidad de la República, Montevideo, Uruguay; ^3^Laboratorio de Fisicoquímica Orgánica, Departamento de Química del Litoral, CENUR Litoral Norte, Universidad de la República, Paysandú, Uruguay

**Keywords:** ^1^H NMR, metabolomics, phylogenetics, phytotoxins, *Streptomyces*

## Abstract

Actinomycetes are generally recognized as a diverse group of gram-positive, mycelium-forming, soil bacteria that play an important role in mineralization processes and plant health, being *Streptomyces* the most well-known genus from this group. Although plant pathogenicity is a rare attribute in this genus, some species have significant impact worldwide due to their ability to cause important crop diseases such as potato common scab (CS). In this work, an integrative approach was applied to investigate the pathogenic potential of *Streptomyces* spp. isolates obtained from a local collection of actinomycetes isolated from potato fields. Secretion of phytotoxic compounds was verified in most pathogenic strains from our collection (27 out of 29), and we followed metabolomic analysis to investigate those phytotoxins. We first evaluated the production of the known phytotoxins thaxtomin A (TXT) and desmethylmensacarcin (DMSN) in phytotoxic *Streptomyces* spp. by HPLC analysis, resulting in 17 TXT and 6 DMSN producers. In addition, NMR-based metabolomic models were able to classify strains according to their phytotoxicity, and metabolomic data was also used to infer chemotaxonomy within pathogenic species. A correlation between phylogeny and the production of distinct phytotoxins was found, supporting the idea that there are “species specific” metabolites produced by this genus. The recently discovered polyketide DMSN was associated unequivocally with *S. niveiscabiei* strains and was not produced by other species in the growth conditions employed. Two *S. niveiscabiei* and two *S. puniciscabiei* phytotoxic strains that did not produce TXT nor DMSN suggest the production of other kind of metabolites involved in phytotoxicity, and allowed the prioritization of these strains for further chemical studies. Indeed, we found two *S. niveiscabiei* strains whose supernatants were not phytotoxic in the radish assay, suggesting other pathogenic mechanisms involved. We believe our work will be useful to help understand relations between metabolites and phylogenetic clades within actinomycetes.

## Introduction

As producers of two-thirds of all known antibiotics and many other bioactive compounds, actinomycetes are a rich source of secondary metabolites. The majority of these compounds are produced by members of the genus *Streptomyces* (Berdy, 2005), which comprises Gram-positive, filamentous, spore-forming actinobacteria that are naturally found in soil and aquatic sediments. It is presumed that in their natural environment, *Streptomyces* produce these metabolites as a strategy for the acquisition of nutrients, to control the growth of competitors, or for signaling and intercellular communication. The ability to infect living plant tissues and to cause diseases of root and tuber crops such as potato common scab (CS) is a rare attribute among members of this genus (Li et al., 2019). Only a dozen or so out of the hundreds of species described up to date, are known to be pathogenic to plants (Loria et al., 2006). The first described and best characterized pathogen species is *S. scabies*, while more recently emerged or restricted pathogens include *S. acidiscabies*, *S. turgidiscabies*, *S. europaeiscabiei*, *S. luridiscabiei*, *S. puniciscabiei*, *S. niveiscabiei*, *S. reticuliscabiei*, and *S. bottropensis* (Lambert and Loria, 1989a,b; Miyajima et al., 1998; Bouchek-Mechiche et al., 2000; Park et al., 2003a; Madhaiyan et al., 2020).

Research on plant-pathogenic *Streptomyces* spp. over the last 10 years has provided vital clues for understanding how these organisms are able to colonize and infect a living plant host and cause disease. In a recent review, known or potential virulence factors have been organized in three categories, including phytotoxins, phytohormones and secreted proteins (Li et al., 2019). Among them, phytotoxins are recognized as key pathogenicity determinants in *Streptomyces*, being thaxtomins the first ones reported and most widely distributed (King et al., 1989; Lawrence et al., 1990; Bignell et al., 2014). More recently, other phytotoxic compounds were reported, including the coronafacoyl phytotoxins and the concanamycins (Natsume et al., 1998, 2018; Bignell et al., 2018). In addition to phytotoxins, many plant pathogens produce phytohormones, such as cytokinins, auxin, and ethylene, as a way to alter hormone signaling in the host, secrete proteins into host plant cells in order to promote pathogenesis, or produce extracellular enzymes that break down plant cell wall polymers to acquire nutrients and to penetrate and spread through host tissues (Li et al., 2019).

An exhaustive identification and genetic characterization of *Streptomyces* spp. responsible for CS in Uruguay was carried out by our group in response to a severe outbreak which caused losses of around 6 million dollars corresponding to nearly one third of the total potato production in 2010 (Lapaz et al., 2017). This study led to a local collection of 331 actinomycetes strains from soil and potato tubers, containing 70 pathogenic *Streptomyces* spp. strains belonging to different species: *S. scabies*, *S. acidiscabies*, *S. europaeiscabiei*, and S. *niveiscabiei.* Using traditional chemical methods, we recently identified and characterized the polyketide desmethylmensacarcin (DMSN) in cultures of a single *S. niveiscabiei* strain (St1015). The pure compound was shown to induce deep necrotic lesions on potato tuber tissue and to cause stunting of radish seedlings (Lapaz et al., 2018). This result was the first report of this compound as a phytotoxin causing CS, and it is not known if it is produced by other *Streptomyces* spp.

While a similar strategy to the one employed in the identification of DMSN could be followed to investigate the remaining pathogenic *Streptomyces* spp. in our local collection, those classical approaches based solely on taxonomical or activity assays often lead to the isolation of previously known compounds. Indeed, bioprospecting of microbial strains for the isolation of active compounds has moved toward integrated strategies that can quickly differentiate new from known metabolites (Betancur et al., 2017). As it facilitates the identification of patterns or metabolite markers that are characteristic of a certain condition and also allows for the comparison of secondary metabolites between species, metabolomic analysis has become an important tool in these type of studies (Nguyen et al., 2016; Betancur et al., 2017). This methodology, which has been applied successfully to a wide range of biological systems (Kim et al., 2011), is particularly useful to obtain information regarding the chemical composition of active extracts prior to compound isolation (Betancur et al., 2017). Metabolomics-based workflows serve as the complementary tool set to compare and contrast genomics-derived expectations with the reality of secondary metabolite production (Hoffmann et al., 2018).

Based on these premises, an integrative approach was applied to investigate the pathogenic potential of our local collection of *Streptomyces* spp. isolates. We initially verified that pathogenicity in most of these strains is due to the secretion of phytotoxic compounds. In addition, the production of the known phytotoxins thaxtomin A (TXT) and desmethylmensacarcin (DMSN) was exhaustively studied in all pathogenic *Streptomyces* spp. Finally, NMR-based metabolomic models capable of classifying strains according to their pathogenicity and in agreement with their phylogeny were developed. As detailed in the following sections, the approach allowed for the identification of two *S. niveiscabiei* and two *S. puniciscabiei* pathogenic strains that do not produce TXT or DMSN, suggesting the secretion of novel bioactive natural products.

## Materials and Methods

### Bacterial Strains and Culture Conditions

A total of 58 actinomycetes strains from our local collection were selected for phytotoxicity evaluation and metabolomic analysis including 29 pathogenic and 29 non-pathogenic strains. Pathogenicity was evaluated in a previous study based on the ability of strains to induce necrosis on potato tuber disks and stunting of radish seedlings (Lapaz et al., 2017). The 29 pathogenic strains included representative isolates of *Streptomyces* species causing common scab in Uruguay (6 *S. scabies*, 6 *S. acidiscabies*, 2 *S. europaeiscabiei*, 9 *S. niveiscabiei*, and 2 *S. puniciscabiei)*, and reference strains for these species (DSM41658, DSM41668, DSM41802, and St1015) (Table 1). In addition, 29 non-pathogenic strains were selected from our local collection and were further identified to the genus/species level in this study (see section “Phylogenetic Analysis of Strains”). All strains were routinely grown on ISP4 agar media at 28°C (Shirling and Gottlieb, 1966), and stored as spore suspensions in 20% glycerol at −70°C. For phytotoxicity, NMR-based metabolomic and HPLC analyses, strains were grown on oat bran broth (OBB) medium (Shirling and Gottlieb, 1966). Briefly, a spore pre-inoculum was prepared in Tween 0.0001% and adjusted to obtain an OD_600_ = 0.1. A 50 μL aliquot of this spore suspension was used to inoculate 50 mL of OBB medium in 250 mL flasks and incubated for 7 days in an orbital shaker at 150 rpm. Cells were then removed by centrifugation at 11,000 × *g* for 10 min followed by filtration (0.45 μm), and the resulting cell-free supernatant used for analyses.

**TABLE 1:** List of 29 pathogenic *Streptomyces* strains used in this study, its phytotoxicity, identification, and detection of known phytotoxic compounds.

			Chemical detection (HPLC)		Genetic detection (PCR)	
Strain ID	Pathogenicity^*a*^/Phytotoxicity	Identification	TXT	DMSN	*txtA*	*dmsnK1*
DSM 41658^T^	+/+	*S. scabies*	+	−	+	−
MAI 2294^b^	+/+	*S. scabies*	+	−	+	−
St124	+/+	*S. scabies*	+	−	+	−
St127	+/+	*S. scabies*	+	−	+	−
St129	+/+	*S. scabies*	+	−	+^*c*^	−
St1232	+/+	*S. scabies*	+	−	+	−
St1113	+/+	*S. scabies*	+	−	+^*c*^	−
DSM 41668^T^	+/+	*S. acidiscabies*	+	−	+	+
St103	+/+	*S. acidiscabies*	+	−	+	+
St105	+/+	*S. acidiscabies*	+	−	+^*c*^	+
St106	+/+	*S. acidiscabies*	+	−	+	+
St113	+/+	*S. acidiscabies*	+	−	+	+
St114	+/+	*S. acidiscabies*	+	−	+	+
St116	+/+	*S. acidiscabies*	+	−	+	+
DSM 41802^T^	+/+	*S. europaeiscabiei*	+	−	+	−
St1140	+/+	*S. europaeiscabiei*	+	−	+	−
St1229	+/+	*S. europaeiscabiei*	+	−	+^*c*^	−
St1015	+/+	*S. niveiscabiei*	−	+	−	+
St107	+/+	*S. niveiscabiei*	−	+	−	+
St108	+/−	*S. niveiscabiei*	−	−	−	−
St109	+/+	*S. niveiscabiei*	−	+	−	+
St1011	+/+	*S. niveiscabiei*	−	−	−	+
St1013	+/+	*S. niveiscabiei*	−	+	−	+
St1016	+/+	*S. niveiscabiei*	−	+	−	+
St1017^b^	+/+	*S. niveiscabiei*	−	+	−	+
St1018^b^	+/+	*S. niveiscabiei*	−	−	−	+
St1020	+/−	*S. niveiscabiei*	−	−	−	+
St1135^b^	+/+	*S. puniciscabiei*	−	−	−	−
St1218	+/+	*S. puniciscabiei*	−	−	−	−

*^*a*^Pathogenicity was determined in a previous study (Lapaz et al., 2017). ^*b*^These strains were added to the previous MLSA scheme (Lapaz et al., 2017). ^*c*^Confirmed by sequencing and Blastn. ^*T*^Type strain.*

### Radish Phytotoxicity Bioassay

The radish seedlings bioassay described by Fyans et al. (2016) was followed with some modifications. Germinated radish seeds were placed into 13 mm diameter wells in 1.5% agar-water plates and then 200 μL of cell-free supernatant were added to each well. A total of five germinated seeds were assayed with each supernatant, and the 58 actinomycetes strains were evaluated at least twice in independent experiments. The plates were wrapped with parafilm and incubated at 24°C under a 16 h photoperiod for 6 days. OBB medium was used as negative control. Root growth inhibition was observed and registered.

### HPLC Analyses

HPLC analysis of TXT and DMSN in cell-free supernatants of the 29 pathogenic *Streptomyces* strains were performed on a Shimadzu Prominence LC-20AT equipped with an SPD-M20A diode-array detector and a Supelco Discovery C-18 column (4.6 × 150 mm, 5 μm particle size). TXT was extracted from 1 mL of cell-free supernatant using Discovery C18 SPS cartridges, and the extracts obtained were loaded onto the HPLC column as described in Johnson et al. (2007). For DMSN detection, 10 mL of the freeze-dried culture supernatants were resuspended in 1 mL of methanol, filtered and loaded onto the HPLC column. Samples were analyzed directly by HPLC using an acetonitrile gradient as described by Yan et al. (2012). Spectra were observed at 380 nm for TXT and at 254 nm for DMSN detection. Both phytotoxic compounds were identified by comparison of their retention times and UV spectra to those of the corresponding standards.

### Phylogenetic Analysis of Strains

Genomic DNA was extracted according to Sambrook and Russell (2001). RNA polymerase beta subunit (*rpoB*) partial gene sequence (Guo et al., 2008) was used as a phylogenetic marker for taxonomic characterization of 29 non-pathogenic actinomycetes strains using primers and conditions described in Lapaz et al. (2017). Briefly, amplified DNA fragments of *rpoB* gene were separated on 1.5% agarose gels and visualized with Good View under UV light. Amplicons were purified and sequenced in both directions by Macrogen Services (Korea). Sequences were assembled, edited and aligned using Geneious 8.0 software (Biomatters, New Zealand). Phylogenies were inferred by the neighbor-joining method (Saitou and Nei, 1987) using partial sequences of 540-bp. Further sequences obtained from the ARS *Streptomycetaceae* MLSA website^1^ were also included in the analysis. The resultant neighbor-joining trees and topology were evaluated using 1000 bootstrap resamplings (Hillis and Bull, 1993). A phylogenetic analysis of pathogenic *Streptomyces* strains used in this study was previously reported (Lapaz et al., 2017) and the correspondent species assignment is shown in Table 1.

### Molecular Detection of DMSN and TXT Specific Genes

Extracted DNA from pathogenic strains was used for amplification of *txtA* gene (which codes for a synthetase involved in thaxtomin A biosynthesis) and *dmsnK1* gene (which codes for a polyketide synthase involved in desmethylmensacarcin biosynthesis). For amplification of *txtA* gene, primers used were *txtA_F* and *txtA_R* (Chapleau et al., 2016), and for *dmsnK1* amplification, the specially designed primers *dmsnK1_F* (GTCCTTCTGGCAGCTCATCT) and *dmsnK1_R* (CGATCATCGACTTGATGGAG) were employed. Amplification of both genes was performed using 25 μL reaction mixtures containing 1X Taq buffer, 1.5 mM of MgCl_2_, 200 μM of each dNTP, 0.5 μM of each primer, 10% v/v of DMSO, 1.0 U of Taq polymerase (Invitrogen), and 20 ng of DNA template. Amplifications were performed in a Thermal Cycler (Applied Biosystems), and the temperature program included an initial denaturation at 94°C for 3 min, 30 cycles of denaturation (95°C and 45 s), annealing (55°C for *dmsnK1* and 65°C for *txtA*, 60 s), and extension (72°C and 90 s), and a final extension at 72°C for 10 min. PCR products were checked by electrophoresis in 1.0% agarose gel with 0.5 μg/mL of GoodView (SBS Genetech Co) in 1X Tris-borate-EDTA (TBE) and visualized under UV light. Selected amplicons were sequenced with reverse and forward primers by Macrogen Services (Korea), assembled and edited using Geneious 8.0 software (Biomatters, New Zealand), and finally compared in GenBank by the Blastn tool.

### NMR Spectroscopy

Cell-free supernatants (10 mL) of the 58 actinomycetes strains were lyophilized, and the resulting dried samples were reconstituted in 600 μL of deuterated water containing 0.05 mg/mL TSP as internal reference. Following centrifugation at 1,500 × *g* for 2 min to remove insoluble particles, the solutions were transferred to 5 mm NMR tubes (NE-HL5-7, New Era Enterprises Inc., United States). Water-suppressed ^1^H NMR spectra were recorded at 25°C on a Bruker AVANCE III 500 NMR spectrometer operating at a ^1^H frequency of 500.13 MHz and equipped with a *z-*gradient TXI probe. A spectral width of 10 KHz, a data size of 32 K, and a total of 64 scans with a relaxation delay of 5 s between scans were employed to record each spectrum.

All ^1^H NMR spectra were manually phased and baseline-corrected using MNova (version 11.0, MestreLab Research, S.L., Spain). Selected regions of the spectra were then aligned by the interval correlation-shifting method (Savorani et al., 2010), and data was normalized to the total spectral area. The region between 3.0 and 5.4 ppm, which includes carbohydrate and residual water resonances, was treated as a blind region and not considered in the analyses. The resulting data matrix was then exported as a text file for use in multivariate analyses.

### Multivariate Statistical Analysis

Principal component analysis (PCA), orthogonal partial least squares discriminant analysis (OPLS-DA), and hierarchical cluster analysis (HCA) were carried out with the PLS_ToolBox package (version 8.5, Eigenvector Research Inc., Manson, WA, United States) as implemented for MATLAB (revision 2015a, The MathWorks Inc., Natick, MA, United States). For all models, data was mean-centered and scaled using a Pareto factor (Van Den Berg et al., 2006). First, data was analyzed performing a PCA, which allows for the identification of strong outliers, reduces the dimensionality and facilitates the identification of data clusters or trends (Wold et al., 1987; Trygg and Wold, 2002; Trygg et al., 2007). Cross-validation of OPLS-DA models was achieved using the random subsets method, which involved 20 iterations over data split into 8 equally sized parts. Receiver operating characteristic (ROC) curves were plotted, and areas under the curves (AUCs) were computed to ensure the goodness-of-fit of the resulting models (Ekelund, 2012; Simundic, 2012). A permutation test with 200 iterations was also performed to determine the degree of over-fitting and further validate the discriminant analyses (Ni et al., 2008). HCA was carried out using Ward’s method (Ward, 1963).

## Results

### Most *Streptomyces* Pathogenic Strains Showed Phytotoxic Activity in Culture Supernatants

**FIGURE 1:**
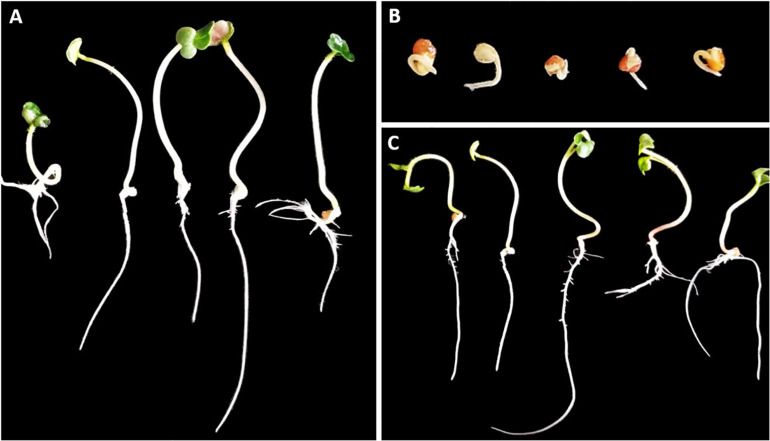
Representative results of the phytotoxicity assay in radish seedlings 6 days after inoculation with cell-free supernatants of 58 actinomycetes strains. Control inoculated with OBB medium **(A)**. Representative *Streptomyces* strains shown as examples of phytotoxic **(B)** and non-phytotoxic **(C)** culture supernatants (strains St107 and MAI2306, respectively). Inoculation with phytotoxic supernatants inhibits root elongation and leads to stunting of shoots, with no significant differences among strains.

Our first objective was to verify that pathogenicity of the selected strains was due to secretion of phytotoxic compounds rather than other mechanisms. The phytotoxicity assays were performed successfully within the 58 strains using OBB medium. Phytotoxicity was confirmed by the complete cessation of root emergence as well as stunted growth of radish shoots after 6 days of incubation (Figure 1).

Twenty-nine non-pathogenic strains were analyzed as non-phytotoxic controls to discard possible phytotoxic effects from other metabolites not associated with pathogenicity produced under those growth conditions. Phylogenetic analysis of these non-pathogenic isolates revealed great diversity, mostly related to at least 16 different *Streptomyces* species (Supplementary Table 1 and Supplementary Figure 1). In addition, five isolates were assigned to *Kitasatospora* sp., other genera of actinomycetes highly related to *Streptomyces*. As expected, those strains did not show phytotoxic activity under the culture conditions used.

On the other hand, most of the pathogenic *Streptomyces* strains showed phytotoxic activity in culture supernatants, verifying that secretion of secondary phytotoxic metabolites is involved in pathogenicity. Only two pathogenic strains identified as *S. niveiscabiei* (St108 and St1020) did not show phytotoxic activity. The pathogenicity of both isolates was previously verified based on the ability to induce root growth inhibition and stunting of shoots in the radish assay, using an inoculum of the strains instead of supernatants (Lapaz et al., 2017). These findings suggest that these strains do not produce phytotoxic compounds under these culture conditions but may do so upon interaction with the plant host.

### Connecting Phytotoxic Activity With Known Phytotoxins Produced by *Streptomyces*

**FIGURE 2:**
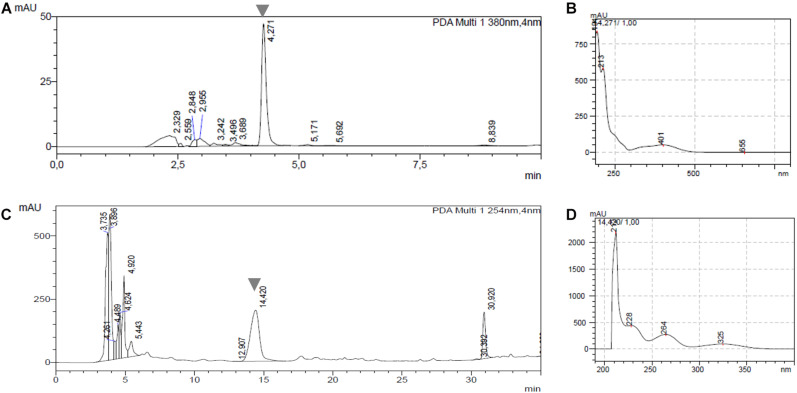
Representative chromatograms and UV-spectra showing detection of thaxtomin A (TXT) and desmethylmensacarcin (DMSN) in cell-free supernatants of 29 pathogenic *Streptomyces* strains. **(A)** Chromatogram from *S. acidiscabies* St105 at 380 nm with the TXT peak at 4.2 min; **(B)** UV spectrum for TXT at 4.2 min; **(C)** chromatogram from *S. niveiscabiei* St1017 at 254 nm showing the DMSN peak at 14.4 min; **(D)** UV spectrum for DMSN at 14.4 min.

As most pathogenic strains from our collection produce phytotoxic compounds, a correlation between this phytotoxic activity and known phytotoxins produced by *Streptomyces* species was assessed. In this context, the production of TXT and DMSN in the 29 pathogenic strains was analyzed by HPLC. Two representative chromatograms for detection of both compounds are shown in Figure 2. The peaks at retention time of 4.2 min corresponding to TXT and at 14.4 min corresponding to DMSN are clearly distinguishable in Figures 2A,C, respectively and the identity of each compound was verified by its UV spectrum. TXT was produced by all tested *S. scabies*, *S. acidiscabies* and *S. europaeiscabiei* strains from our collection (Table 1). In addition, DMSN was detected in culture supernatants of 6 of the 10 *S. niveiscabiei* strains, suggesting that, at least within our collection, this recently reported phytotoxin is unique for this pathogenic species. In agreement with the radish phytotoxicity assay, neither of these two phytotoxins were detected in supernatants of strains St108 and St1020. Additionally, we did not detect neither TXT nor DMSN in the four other strains whose supernatants showed phytotoxicity (St1011, St1018, St1135, and St1218).

As a complementary tool to infer about the capability for production of these phytotoxins, detection of *dmsnK1* and *txtA* genes was performed. As expected, the presence of the corresponding biosynthetic genes was confirmed in all isolates producing each phytotoxin. Interestingly, the DMSN target gene, *dmsnK1*, is present in all *S. acidiscabies* isolates, although this phytotoxin was not detected by HPLC. These results may be explained by the existence of silent or truncated biosynthetic gene clusters (BGCs), so production of the phytotoxin is not observed at least in the selected culture conditions. In the case of *S. niveiscabiei* isolates, different results were found. None of the isolates produced TXT nor the *txtA* gene was detected. While *dmsnK1* was detected in almost all *S. niveiscabiei* isolates, only 6 out of 10 isolates produced DMSN (St1015, St107, St109, St1013, St1016, and St1017). These results also suggest that despite this gene being present in the genome, it is not expressed in some cases. Some isolates harboring the *dmsnk1* gene do not produce its phytotoxin in the studied conditions, and, as they showed phytotoxicity this result indicates that these strains may produce other phytotoxins. Finally, *S. puniciscabiei* isolates do not harbor any of these genes and neither of these phytotoxins were detected, suggesting secretion of other unidentified toxins.

### Connecting the Metabolic Profile With Pathogenicity Potential and Phylogeny

**FIGURE 3:**
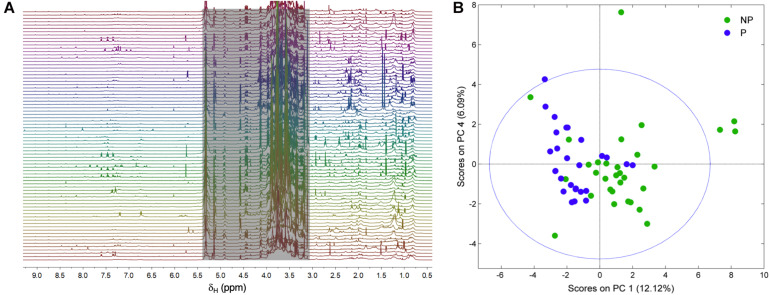
Stacked ^1^H NMR spectra from the supernatants of the 58 actinomycetes strains analyzed in this study **(A)**. Data from the grayed-out region ranging from 3.0 to 5.4 ppm, including signals from carbohydrates and residual water, was not considered in the multivariate analyses. PCA score plot obtained from ^1^H NMR spectral data showing clustering of phytotoxic (P) and non-phytotoxic (NP) supernatants **(B)**.

**FIGURE 4:**
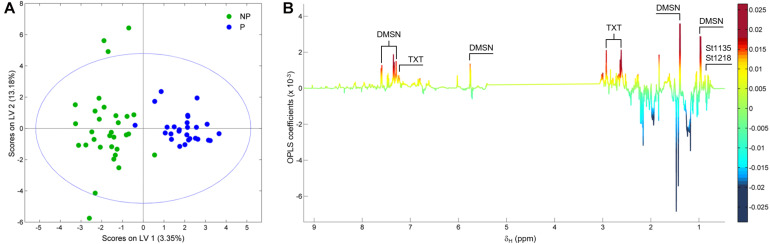
Score **(A)** and loading factor **(B)** plots obtained from the OPLS-DA between 27 phytotoxic (P) and 31 non-phytotoxic (NP) *Streptomyces* strains. Signals corresponding to metabolites that correlate with phytotoxic strains are annotated in the loading factor plot. The model had R^2^Y and Q^2^Y coefficients of 0.81 and 0.54, respectively, and a ROC curve with an AUC of 0.93. A permutation test performed with 200 iterations fulfills the Wilcoxon test with *p* < 0.05 (see Supplementary Figure 2).

Figure 3A shows the ^1^H NMR spectra from the supernatants of the 58 strains employed in this study. As stated earlier, the grayed-out region ranging from 3.0 to 5.4 ppm contains mostly resonances from carbohydrates that make up the growth medium and the residual solvent peak, and it was therefore not considered in the analysis. Variations in the exometabolome ^1^H NMR profiles can be readily observed in both the aromatic-olefinic (9.0 to 5.5 ppm) and aliphatic (3.0 to 0.0 ppm) regions throughout the spectra, and these are the basis for the development of models to discriminate between phytotoxic and non-phytotoxic *Streptomyces* strains based on multivariate analyses. A PCA was carried out initially to evaluate data structure, and the resulting score plot is presented in Figure 3B. Two clusters that group phytotoxic and non-phytotoxic strains can be distinguished, so we therefore constructed a statistical model capable of discriminating between the two groups by means of an OPLS-DA (Figure 4A). The validated model showed good discrimination between both groups (see Supplementary Material). Cursory inspection of the OLPS-DA loading plots shown in Figure 4B, indicates that supernatants from pathogenic strains correlate with signals corresponding to the known phytotoxins including TXT (7.29, 2.93, and 2.62 ppm) and DMSN (7.60, 7.46, 7.34, 5.76, 1.39, and 0.97 ppm), as well as resonances at 0.88 and 0.77 ppm that can be traced back unequivocally to the ^1^H profiles from *S. puniciscabiei* strains St1135 and St1218.

**FIGURE 5:**
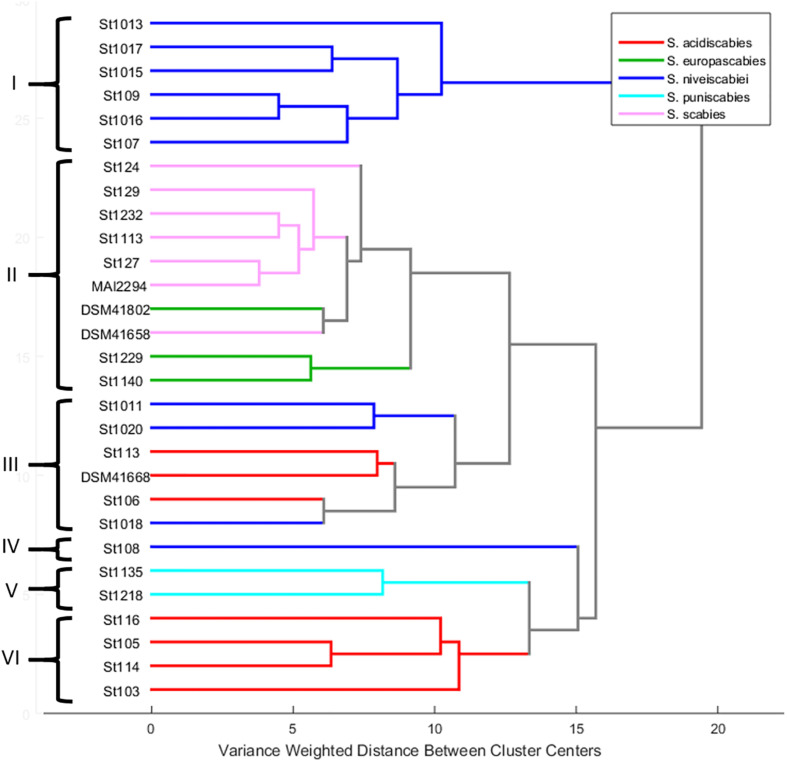
HCA performed by Ward’s method using mean-centered and Pareto-scaled ^1^H NMR data of the 29 pathogenic strains. Roman numerals represent clusters according to their metabolic profile similarity, and different *Streptomyces* spp. are color-coded as indicated in the legend.

An HCA was carried out considering only the 29 pathogenic strains of our collection. The HCA dendrogram presented in Figure 5 shows that supernatants were grouped into six clusters (I to VI) according to their ^1^H NMR profile. If these results are compared with those obtained from the MLSA several similarities between both classification approaches are observed (Table 1). For instance, all strains identified as *S. scabies* and *S. europaeiscabiei* were grouped in cluster II with a little difference between them, as observed in MLSA where both species are closely related phylogenetically. Additionally, all strains from cluster I belong to the species *S. niveiscabiei*, although some other strains of this species were grouped in clusters III and IV (St108, St1011, St1018, and St1020). The large distance from cluster I relative to other clusters (clusters II-VI) suggests important differences in terms of metabolic profiles which can be explained by the production of DMSN by those strains. In ^1^H NMR profiles from these DMSN-producing strains, the resonances previously reported for this metabolite can be observed in Supplementary Figure 3 (Lapaz et al., 2018). The same situation can be observed within strains of species *S. acidiscabies*, which were grouped in clusters III and VI. Finally, strains grouped in cluster V were phylogenetically identified as *S. puniciscabiei*.

## Discussion

Few studies have examined differences in secreted metabolites within plant-pathogenic *Streptomyces* spp. strains. In this work, this issue was addressed from a metabolomic and phylogenetic perspective. For several years, thaxtomins have been identified as essential pathogenicity determinants within plant-pathogenic *Streptomyces* species. However, the great divergence of this genus and its high metabolic potential, added to its ability to mobilize key biosynthetic genes clusters by horizontal gene transfer, lead to the emergence of new pathogenic species (Huguet-Tapia et al., 2016; Zhang et al., 2016). Recent studies have shown that other phytotoxic compounds also contribute to the development of CS, like some specialized metabolites that were not previously known to exhibit phytotoxicity (Li et al., 2019). To delve into these findings, in this work we followed an NMR-based metabolomic approach to determine the pathogenic potential of *Streptomyces* strains and, eventually, identified new strains having the newly discovered phytotoxin or other phytotoxins.

Our local collection of 331 actinomycetes strains from soil and potato tubers includes 70 plant-pathogenic *Streptomyces* spp. strains belonging to different species. The remaining non-pathogenic strains isolated in the same survey were not previously studied. The high diversity found in our phylogenetic analysis reveals the heterogenicity of this group of bacteria (Supplementary Figure 1). Furthermore, our study shows that phylogenetic analyses based on *rpoB* can be used as a method of differentiating unknown isolates, although further studies will be carried out to determine the precise taxonomical identification at the species level.

A radish assay previously used to infer pathogenicity was adapted and applied in this work to infer phytotoxicity of supernatants (Bignell et al., 2010; Fyans et al., 2016). As expected, almost all supernatants obtained from pathogenic strains were active in this assay, indicating that pathogenicity of these *Streptomyces* strains is explained by the secretion of secondary metabolites and/or proteins with phytotoxic activity. Results obtained for 29 non-pathogenic strains showing non-phytotoxic activity allowed us to validate the interpretation of positive results, excluding the existence of phytotoxic compounds derived from degradation of media substrates during bacterial growth. Results of radish assay using pure TXT and DMSN as positive controls were previously confirmed (Lapaz et al., 2018).

In a recent review, Li et al. (2019) provide an overview of current knowledge regarding virulence factors contributing to host infection and disease development by pathogenic *Streptomyces* spp. Apart from thaxtomins, there is now evidence that other phytotoxins play a key role in the development or severity of plant diseases caused by *Streptomyces* spp. Previous studies from our group lead to the discovery of DMSN as a new phytotoxic compound produced by *S. niveiscabiei* strain St1015 (Lapaz et al., 2018). In addition, many others phytotoxins were reported, including the coronafacoyl (Bignell et al., 2010, 2018), concanamycins (Fyans et al., 2016), borrelidin (Cao et al., 2012), and fridamycin E (Natsume et al., 2018). In this work, we hypothesized that DMSN and/or TXT might be present in several strains from our collection, so we performed HPLC analysis to detect them in culture supernatants, as well as PCR detection of key genes of its metabolic pathway involved. Our results showed that TXT was produced by all tested *S. scabies*, *S. acidiscabies* and *S. europaeiscabiei* isolates from our collection (Table 1), and as expected, the presence of *txtA* gene was confirmed in those strains. Indeed, the *txtA* gene was absent in all tested *S. niveiscabiei* strains, whereas the presence of this gene is reported in the type strain of this species; NRRL B-24457 (Park et al., 2003a). We suggest that these strains might have lost this gene or BGC, as has been observed with some strains from different species (*S. scabies* and *S. acidiscabies)* (Zhang et al., 2016, 2018).

Interestingly, apart from detecting the presence of DMSN in strain St1015, which was used as reference strain, it was detected in culture supernatants of other five *S. niveiscabiei* strains. It seems that, at least within our collection, this recently reported phytotoxin is unique and characteristic for this pathogenic species. These findings are similar to those obtained by other authors, for example, the production of concanamycins seem to be particular to *S. scabies* (Natsume et al., 1998, 2001). Recently, *S. stelliscabiei*, a pathogenic species causant of CS in potato, was reclassified as *S. bottropensis* (Madhaiyan et al., 2020), from which DMSN was isolated for the first time (Arnold, 2002). We demonstrate that *S. niveiscabiei* is a novel species associated with CS that produces this phytotoxin. Further studies involving exploration by bioinformatic analysis of the corresponding BGC in other *Streptomyces* species is being performed by our group. Interestingly, *dmsnK1* (a key gene in the DMSN metabolic pathway) was detected in all *S. acidiscabies* and almost all *S. niveiscabiei* strains of our collection (except St108). The fact that this toxin is not produced by *S. acidiscabies* strains harboring the *dmsnK1* gene can be explained by the existence of silent or truncated BGCs, or by culture conditions used in this study. OBB medium was selected in this work as it is reported that all known phytotoxins are induced under those conditions (Babcock et al., 1993; Natsume et al., 1996, 1998, 2005, 2018; Cao et al., 2012; Fyans et al., 2015). Despite this, regulation mechanisms involved may be complex, as secondary metabolites are not constitutively expressed. The specific conditions required for its expression make their study difficult in the laboratory.

We found out that for two *S. niveiscabiei* strains (St1011 and St1018) showing phytotoxicity, neither DMSN nor TXT were detected by HPLC. Indeed, other two strains, St1135 and St1218, related to *S. puniciscabiei* in the MLSA scheme, did not produce any of the known compounds and do not harbor the corresponding genes either. The culture supernatants of these strains were able to produce root growth inhibition and stunting of shoots in the radish assay, not differing from TXT- or DMSN-producing strains. For these strains, we propose the production of other phytotoxic compounds which can be novel. Up to now, no phytotoxin has been reported for *S. puniciscabiei* (Park et al., 2003b).

To evaluate differences in metabolite production, a metabolomic approach based on ^1^H NMR analysis was carried out. The high amount of data used in metabolomics requires multivariate data analysis to classify the samples into different groups and to facilitate their interpretation in terms of metabolite distribution under distinct variables. It also allows to simultaneously evaluate many metabolites and determine their correlations with certain biological properties (Betancur et al., 2017). Metabolomics has traditionally been used in humans and plants (Nicholson and Lindon, 2008; Kim et al., 2011), but is less used in microbes. In actinomycetes, most reports made on metabolomics are focused on identification of new compounds with biological activity like antibiotics rather than other bioactive metabolites (Betancur et al., 2017; Wu et al., 2017; Senges et al., 2018), and most of them use LC-MS/MS. In this work, we use NMR-based metabolomics as a tool for exploring the pathogenic potential of this genus. We found that comparison between phytotoxic and non-phytotoxic strains by an OPLS discriminant analysis showed different chemical profiles. The corresponding loading plots showed that discrimination was mostly due to peaks corresponding to known compounds (DMSN and TXT), and a putative phytotoxin associated with *S. puniciscabiei* strains St1135 and St1218 that remains to be identified. On the other hand, signals from non-phytotoxic strains do not show a specific pattern and were mostly related to primary metabolites, including lipoproteins and *N*-acetylglucosamine.

Despite the gradually increasing number of reports related to phylogenetics or metabolomics over the past years, few of them have reported metabolomic studies from a phylogenetic perspective. Nguyen et al. (2016) relate phylogenetics studies to MS-based metabolomics using stepwise partial least squares-discriminant analysis (PLS-DA) for the plant genus *Panax*, revealing the relationships between metabolic changes and the evolutionary adaptations of the species to different climates. Regarding actinobacteria, Betancur et al. (2017) used an integrative strategy based on taxonomic information, bioactivity and metabolic profiling tools, along with dereplication procedures, to prioritize strains as a source of novel and active compounds against phytopathogens. Here, we compared results obtained from the MLSA phylogenetic tree to an HCA dendrogram derived from metabolic profiles based on ^1^H NMR within pathogenic *Streptomyces* species to investigate the relation between taxonomy and the production of phytotoxic compounds. In HCA, the distance between two objects corresponds to the degree of similarity between them. Hence, in this case, a small cluster distance indicates a high similarity between the chemical profiles of the strains, and as this distance increases, so does the degree of divergence among them (Betancur et al., 2017). The large distance from cluster I relative to the other clusters (cluster II-VI), suggests important differences in terms of metabolic profiles which can be explained by secretion of DMSN by those strains (Supplementary Figure 3), all belonging to *S. niveiscabiei*. It seems that the high signal intensities of resonances corresponding to this compound do have an important effect in the discrimination (Figure 4B). This finding highlights the biological importance of DMSN, being a secondary metabolite secreted in large amounts by *S. niveiscabiei* strains. The high relation shown in the HCA by strains identified as *S. scabies* and *S. europaeiscabiei* indicates a close link between phylogenetics and metabolomics in this case. On the other hand, some strains identified as *S. niveiscabiei* (St108, St1011, St1018, and St1020) were grouped in clusters III and IV, and a similar situation can be observed within strains of species *S. acidiscabies* which were grouped in clusters III and VI. The fact that HCA does not match the phylogenetic reconstruction (MLSA in this case) is expected as these techniques focus on different sets of information, making them complementary. Previous studies have demonstrated that different strains from the same *Streptomyces* species contain different gene clusters encoding the production of strain-specific secondary metabolites (Seipke, 2015). This work presents examples of phylogenetically related strains which do not produce the same secondary metabolites, and strains belonging to different clades with similar metabolite production (as observed by strains of *S. acidiscabies* producing thaxtomin). Similar results were found by Betancur et al. (2017) related to active compounds against phytopathogens. Finally, strains grouped in cluster V were phylogenetically related to *S. puniciscabiei*, so phylogenetic and metabolomics are also related in this case and we believe that for these strains a non-identified phytotoxic compound is involved. This study allowed us to prioritize those strains for further chemical studies.

The approach taken here has the potential to directly pinpoint candidates for novel metabolites. This conclusion is supported by the correlation between the metabolome diversification and phylogenetic distance substantiated in this study. We believe that for those four strains (St1011, St1018, St1135, and St1218) belonging to S. *niveiscabiei* and *S. puniciscabiei* in which we did not detect neither TXT nor DMSN, other phytotoxic compounds are present. More importantly, these four strains would have been classified as phytotoxin-producers by the PCA, HCA, and OPLS-DA models even if phytotoxicity data had not been available for them, clearly highlighting the time-saving benefits of the proposed methodology.

As a final remark, it should be noted that bacteria producing several compounds (i.e., metabolic rich bacteria) generally produce low amounts of compounds, making their isolation and identification difficult. With both NMR and MS-based detection technologies, the identification of the metabolites in the biological sample remains a significant obstacle and bottleneck (Dona et al., 2016). Public databases for storing and sharing information are still lacking for NMR-based metabolomic analysis. Such databases are urgently needed to make metabolic profiling a really robust “omics” technology (Kim et al., 2011).

## Data Availability Statement

The raw data supporting the conclusions of this article will be made available by the authors, without undue reservation.

## Author Contributions

VC performed all experiments. AL-R and GM contributed to the design of metabolomic experiments and elaboration and interpretation of multivariate data analysis. ML contributed to HPLC analysis. MS contributed to experimental design and data analysis. MP revised the manuscript critically. All authors made substantial and intellectual contributions to the work, wrote the manuscript, and approved it for publication.

## Conflict of Interest

The authors declare that the research was conducted in the absence of any commercial or financial relationships that could be construed as a potential conflict of interest.

## Footnotes

1 http://199.133.98.43/Streptomyces/
