# Supplementary material for: An Integrative Approach for the Characterization of Plant-Pathogenic Streptomyces spp. Strains Based on Metabolomic, Bioactivity, and Phylogenetic Analysis
Source: Front Microbiol. 2021 Mar 22;12:643792. doi: 10.3389/fmicb.2021.643792 (PMC8019742; doi:10.3389/fmicb.2021.643792)
Supplement: Supplementary file 1 [file Data_Sheet_1.docx]

***Supplementary Material***

**Supplementary Table S1.** List of the 58 actinomycetes strains used in this study.

| **Strain ID** | **Origin** | **Pathogenicity ^a^/Phytotoxicity** | **Identification (based on MLSA or *rpoB*)^b^** |
| --- | --- | --- | --- |
| DSM 41658^T^ | Reference strain | +/+ | *S. scabies* |
| MAI 2294 | 2011, San José | +/+ | *S. scabies* |
| St124 | 2012, San José | +/+ | *S. scabies* |
| St127 | 2012, San José | +/+ | *S. scabies* |
| St129 | 2012, San José | +/+ | *S. scabies* |
| St1232 | 2012, San José | +/+ | *S. scabies* |
| St1113 | 2011, San José | +/+ | *S. scabies* |
| DSM 41668^T^ | Reference strain | +/+ | *S. acidiscabies* |
| St103 | 2010, San José | +/+ | *S. acidiscabies* |
| St105 | 2010, San José | +/+ | *S. acidiscabies* |
| St106 | 2010, San José | +/+ | *S. acidiscabies* |
| St113 | 2011, Rocha | +/+ | *S. acidiscabies* |
| St114 | 2011, Rocha | +/+ | *S. acidiscabies* |
| St116 | 2010, San José | +/+ | *S. acidiscabies* |
| DSM 41802^T^ | Reference strain | +/+ | *S. europascabiei* |
| St1140 | 2011, Rocha | +/+ | *S. europascabiei* |
| St1229 | 2012, San José | +/+ | *S. europascabiei* |
| St1015 | Reference strain | +/+ | *S. niveiscabiei* |
| St107 | 2010, San José | +/+ | *S. niveiscabiei* |
| St108 | 2010, San José | +/- | *S. niveiscabiei* |
| St109 | 2010, San José | +/+ | *S. niveiscabiei* |
| St1011 | 2010, San José | +/+ | *S. niveiscabiei* |
| St1013 | 2010, San José | +/+ | *S. niveiscabiei* |
| St1016 | 2010, Canelones | +/+ | *S. niveiscabiei* |
| St1017 | 2010, Canelones | +/+ | *S. niveiscabiei* |
| St1018 | 2010, Canelones | +/+ | *S. niveiscabiei* |
| St1020 | 2010, Canelones | +/- | *S. niveiscabiei* |
| St1135 | 2011, San José | +/+ | *S. puniciscabiei* |
| St1218 | 2012, San José | +/+ | *S. puniciscabiei* |
| MAI2200 | 2016, Córdoba | -/- | *S. baarnensis, S. bohaiensis, S. fimicarius* |
| MAI2203 | 2016, Córdoba | -/- | *S. baarnensis, S. bohaiensis, S. fimicarius* |
| MAI2205 | 2016, Córdoba | -/- | *S. chartreusis* |
| MAI2211 | 2011, San José | -/- | *S. naganishii* |
| MAI2218 | 2012, San José | -/- | *S. laurentii* |
| MAI2220 | 2012, San José | -/- | *S. antibioticus* |
| MAI2221 | 2012, San José | -/- | *S. pseudovenezuelae* |
| MAI2222 | 2012, San José | -/- | *Kitasatospora sp.* |
| MAI2223 | 2012, San José | -/- | *S. bungoensis* |
| MAI2227 | 2011, San José | -/- | *S. flaveolus* |
| MAI2228 | 2011, San José | -/- | *S. naganishii* |
| MAI2230 | 2011, San José | -/- | *Kitasatospora phosalacinea* |
| MAI2231 | 2011, San José | -/- | *S. novaecaesareae* |
| MAI2235 | 2012, San José | -/- | *Kitasatospora atroaurantiaca* |
| MAI2239 | 2012, San José | -/- | *S. lydicus* |
| MAI2281 | 2012, San José | -/- | *S. xanthochromogenes* |
| MAI2282 | 2012, San José | -/- | *S. longisporus* |
| MAI2285 | 2012, San José | -/- | *S. flaveolus* |
| MAI2287 | 2012, San José | -/- | *Kitasatospora setae* |
| MAI2289 | 2012, San José | -/- | *S. pseudovenezuelae* |
| MAI2299 | 2012, San José | -/- | *S. graminisoli* |
| MAI2303 | 2012, San José | -/- | *S. murinus* |
| MAI2306 | 2012, San José | -/- | *S. baarnensis, S. bohaiensis, S. fimicarius* |
| MAI2308 | 2012, San José | -/- | *S. baarnensis, S. bohaiensis, S. fimicarius* |
| MAI2321 | 2012, San José | -/- | *S. rishiriensis* |
| MAI2327 | 2012, San José | -/- | *S. pseudovenezuelae* |
| MAI2336 | 2012, San José | -/- | *S. pseudovenezuelae* |
| MAI2340 | 2012, San José | -/- | *S. nitrosporeus* |
| MAI2402 | 2012, San José | -/- | *S. scopuloiridis* |

**^a^** Pathogenicity was determined in a previous study (Lapaz et al., 2017).

**^b^** Identification within non-pathogenic strains was based on *rpoB* partial gene sequences (540 bp) and the most related species/s resulted from the phylogenetic tree (Figure S1) are shown.


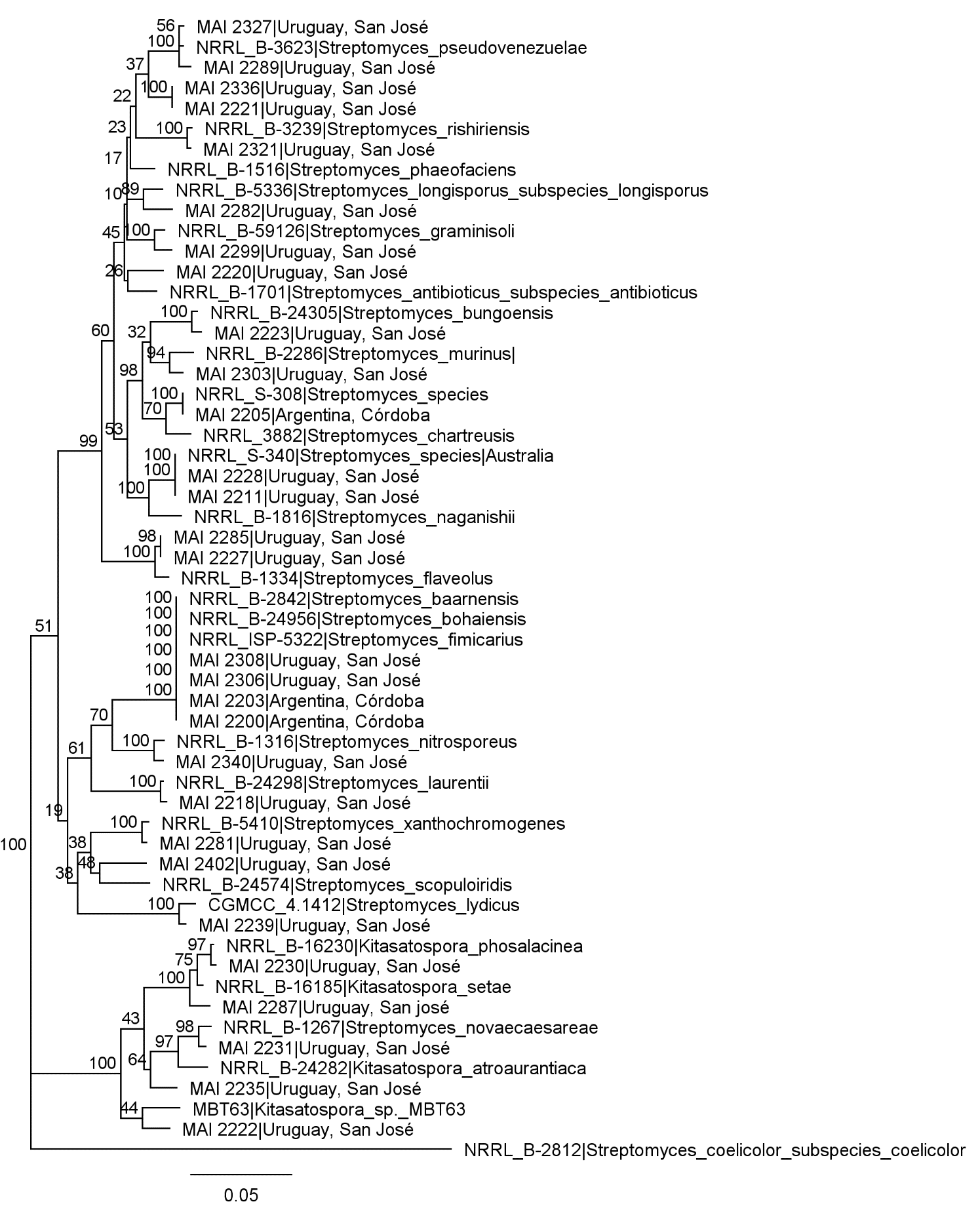


**Supplementary Figure 1.** Neighbor-joining tree of 29 non-pathogenic actinomycetes strains used in this study constructed from *rpoB* partial gene sequences (540 bp). Sequences from additional reference strains were obtained from the ARS *Streptomycetaceae* MLSA website (<http://199.133.98.43/Streptomyces/>).


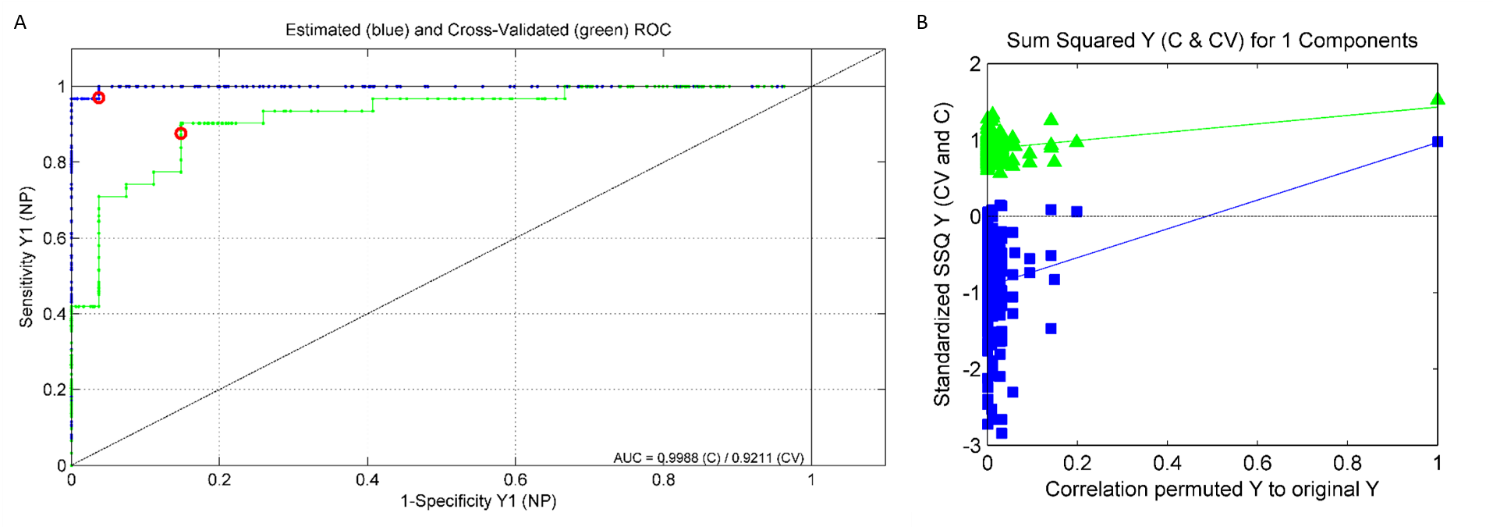


**Supplementary Figure 2.** ROC curves (a) and permutation test (b) obtained from the cross-validation of the OPLS-DA between phytotoxic and non-phytotoxic supernatants.

**
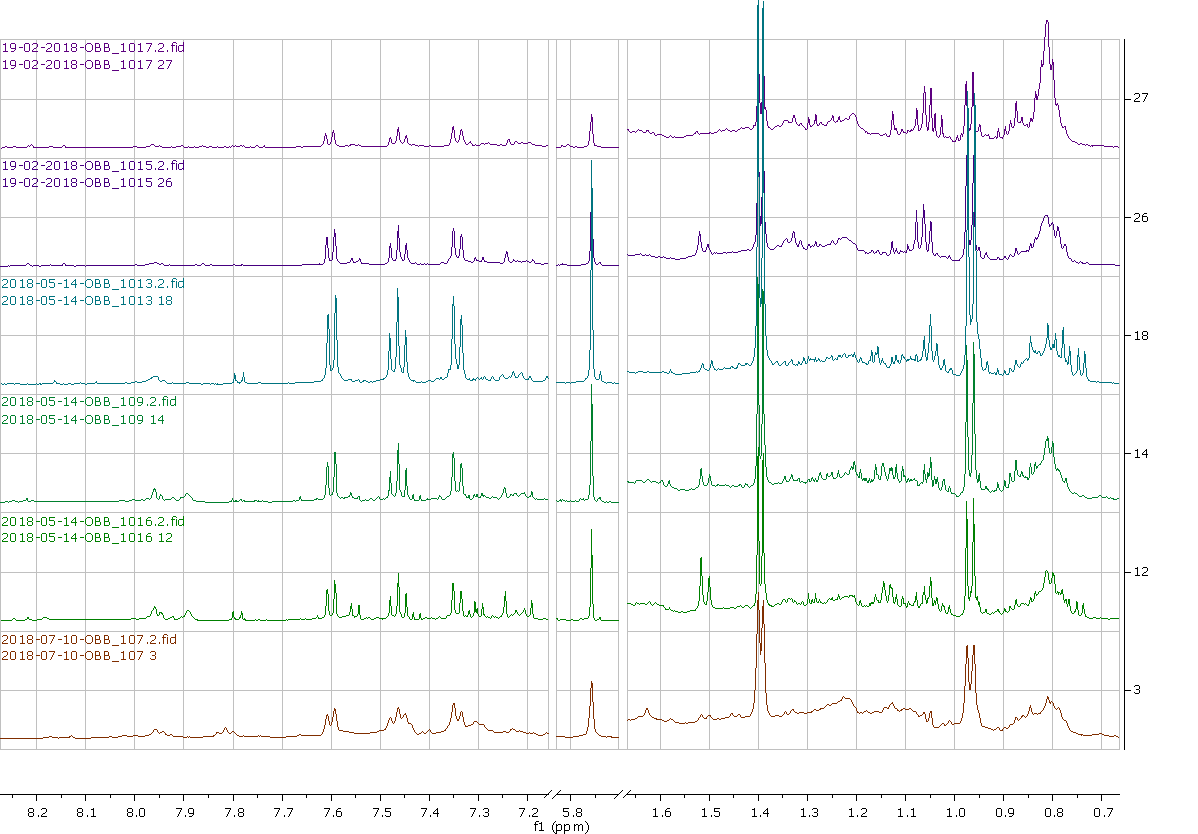
**

**Supplementary Figure 3.** Metabolic H^1^ NMR spectra from supernatants of desmethylmensacarcin (DMSN) producing *Streptomyces niveiscabiei* strains (St1017, St1015, St1013, St109, St1016 and St107). DMSN characteristic peaks (δ_H_=1.06 ppm, δ_H_=1.44 ppm, δ_H_= 5.87 ppm, δ_H_= 7.36 ppm, δ_H_= 7.48 ppm and δ_H_=7.68 ppm) are clearly visible.
